# Gut Microbiota and Thyroid Diseases

**DOI:** 10.5152/eurasianjmed.2026.251232

**Published:** 2026-04-15

**Authors:** Yunus Emre Beyhan, Şeyma Işık

**Affiliations:** 1 Department of Parasitology, Yalova University Faculty of Medicine, Yalova, Türkiye; 2 Department of Medical Parasitology, Yüzüncü Yıl University Faculty of Medicine, Van, Türkiye

**Keywords:** Autoimmunity, dysbiosis, gut microbiota, thyroid disorders

## Abstract

The gut microbiota plays a pivotal role in maintaining the homeostasis of the digestive and immune systems. Dysbiosis compromises the intestinal barrier and triggers systemic inflammation, thereby contributing to metabolic and immune dysregulation. Emerging evidence suggests a bidirectional relationship between the gut microbiota and thyroid function. The microbiota influences the absorption and metabolism of micronutrients essential for thyroid hormone synthesis, including iodine, selenium, iron, and zinc. Dysbiosis enhances intestinal permeability, facilitating the translocation of bacterial components into systemic circulation and potentially triggering autoimmune thyroid diseases (AITD) such as Hashimoto’s thyroiditis and Graves’ disease. Animal studies further demonstrate that hypothyroidism, hyperthyroidism, and thyroidectomy can alter the diversity and composition of gut microbiota. Environmental exposures such as particulate matter 2.5 reshape microbial and metabolic profiles, influencing the hypothalamus–pituitary–thyroid axis and thyroid hormone homeostasis. Clinical studies show reduced abundance of beneficial bacteria (*Bifidobacterium*and*Lactobacillus*) and enrichment of proinflammatory taxa in AITD patients. These findings highlight the complex bidirectional interactions between gut microbiota, thyroid function, and immune tolerance. Microbiota-targeted therapies may represent promising strategies for the prevention and management of thyroid disorders. This review aims to summarize current evidence regarding the bidirectional relationship between gut microbiota and thyroid disorders, with particular emphasis on dysbiosis, immune regulation, micronutrient metabolism, and autoimmune thyroid disease.

**Main Points**

- The gut microbiota directly affects thyroid function and immunity.
- Dysbiosis triggers the development of autoimmune thyroid diseases (Hashimoto’s and Graves’).
- The microbiota regulates the absorption of iodine, selenium, iron, and zinc, which are necessary for thyroid hormone synthesis.
- Environmental factors (e.g., PM2.5) and hormonal disorders alter the microbiota structure.
- Microbiota-based treatments (probiotics, prebiotics, diet, etc.) offer new hope in preventing thyroid diseases.

## Introduction

The gut microbiota plays a critical role in maintaining systemic homeostasis by supporting both digestive and immunological balance. When the microbiota is disrupted (“dysbiosis”), epithelial barrier function is compromised, predisposing to systemic and intestinal inflammation, metabolic dysfunction, and immune dysregulation. Due to its pleiotropic influence on the intestinal barrier, nutrient metabolism, and immune system development and function, the gut microbiota has become a focal point of research interest. Emerging evidence suggests that alterations in gut microbial composition may contribute to the pathogenesis and progression of autoimmune diseases, both within the gastrointestinal tract and at distant sites.^1^ ^-^^3^

Approximately 70% of the human immune system resides in the intestinal mucosa, highlighting the importance of the gut in immune development and tolerance. Consequently, studies have consistently demonstrated associations between dysbiosis and pathological immune responses. Building on this understanding, recent research indicates that thyroid disorders—particularly autoimmune thyroid diseases (AITD)—may be influenced by imbalances in gut microbial communities.^4^

Beyond immune regulation, accumulating evidence indicates that the gut microbiota contributes to thyroid homeostasis by modulating the intestinal handling of key micronutrients. Microbial communities influence the intestinal environment through effects on epithelial integrity, bile acid transformation, and transporter activity, thereby shaping the bioavailability of iodine, selenium, iron, and zinc. These micronutrients are indispensable for thyroid hormone synthesis and activation, particularly selenium and zinc, which are required for deiodinase-mediated conversion of thyroxine to triiodothyronine. Consequently, microbial imbalance may indirectly impair thyroid hormone metabolism by disrupting micronutrient availability and immune equilibrium.^2^^,^^3^

**Figure 1.**
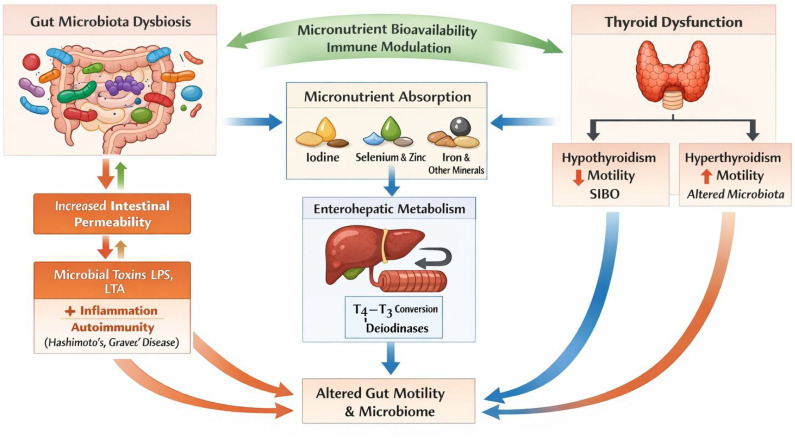
Schematic representation of the bidirectional gut–thyroid axis.

Figure 1 schematically illustrates the bidirectional interactions between gut microbiota, micronutrient metabolism, immune regulation, and thyroid hormone homeostasis.

The gut microbiota regulates thyroid function through modulation of micronutrient bioavailability (iodine, selenium, iron, and zinc), intestinal barrier integrity, immune tolerance, and enterohepatic metabolism of thyroid hormones. Dysbiosis increases intestinal permeability, promotes inflammatory and autoimmune responses such as Hashimoto’s thyroiditis (HT) and Graves’ disease (GD), while thyroid dysfunction reciprocally alters gut motility and microbial composition. The aim of this review is to evaluate current evidence on the gut–thyroid axis and to discuss how alterations in gut microbiota may influence thyroid function, immune tolerance, and the development of autoimmune thyroid disorders.

## Material and Methods

A narrative literature review was conducted using PubMed, Scopus, and Web of Science databases. Publications in English from 2000 to 2025 addressing gut microbiota, thyroid physiology, and autoimmune thyroid disorders were evaluated. Both experimental and clinical studies were considered, with emphasis on original research and mechanistic evidence relevant to the gut–thyroid axis.

### Thyroid Disorders and the Gut–Thyroid Axis

Thyroid hormones are essential for energy homeostasis and regulation of metabolic processes. However, the relationship between thyroid function and host gut microbial communities remains incompletely understood. Microbial disturbances may contribute to the pathogenesis of both gastrointestinal diseases and autoimmune thyroid disorders. The gut microbiota regulates the absorption and metabolism of micronutrients such as iodine and selenium, which are required for thyroid function, as well as for the maintenance of immune tolerance. Dysbiosis may compromise intestinal barrier integrity, allowing bacterial components and toxins to translocate into systemic circulation and initiate autoimmune responses. Hashimoto’s thyroiditis (HT) and GD are key examples of AITD linked to these mechanisms.^5^^-^^7^

Thyroid dysfunction also exerts secondary effects on gastrointestinal physiology, which in turn shape the gut microbial ecosystem. Reduced intestinal motility observed in hypothyroidism may favor bacterial overgrowth, whereas accelerated transit in hyperthyroidism alters microbial diversity patterns. In addition, intestinal microbes participate in the metabolism and recycling of thyroid hormones by modifying iodothyronine conjugates within the enterohepatic circulation. These processes collectively illustrate a dynamic, bidirectional interaction between thyroid hormone status and gut microbial composition.^4^^-^^6^

Beyond micronutrient metabolism, the gut microbiota influences the metabolism of nutrients, drugs, and hormones—including exogenous and endogenous iodothyronines. While dysbiosis is implicated in multiple autoimmune disorders, its role in AITD, the most prevalent autoimmune thyroid condition, remains incompletely elucidated. Moreover, hypothyroidism and hyperthyroidism, which are frequently autoimmune in origin, have been linked to small intestinal bacterial overgrowth (SIBO) and alterations in microbial composition, respectively.^8^^-^^10^

Additionally, gut microbes modulate the bioavailability of essential micronutrients that are indispensable for thyroid physiology, thereby influencing both hormone synthesis and immune regulation. Iodine is a fundamental structural component of thyroxine (T4) and triiodothyronine (T3), and its availability tightly controls thyroid hormone synthesis through autoregulatory mechanisms such as the Wolff–Chaikoff effect; both iodine deficiency and excess can disrupt normal hormonogenesis. Iron and copper are directly involved in thyroid hormone synthesis as essential cofactors for thyroid peroxidase, the heme-dependent enzyme that catalyzes iodide oxidation, organification, and coupling reactions, while also contributing to oxidative balance and mitochondrial function within thyroid follicular cells. In contrast, selenium and zinc play critical roles in peripheral thyroid hormone metabolism and signaling. Selenium is an essential component of iodothyronine deiodinases responsible for the conversion of T4 to the biologically active T3, as well as of antioxidant enzymes such as glutathione peroxidases and thioredoxin reductases that protect thyroid tissue from hydrogen peroxide–induced oxidative stress. Zinc contributes to thyroid hormone receptor structure, transcriptional regulation, modulation of deiodinase activity, and regulation of thyrotropin (TSH) synthesis and signaling. Moreover, vitamin D supports thyroid homeostasis indirectly by modulating immune tolerance and inflammatory signaling pathways, including T-cell differentiation and cytokine production, thereby influencing susceptibility to autoimmune thyroid dysfunction.^5^^,^^6^^,^^8^^,^^10^ Deficiencies in these micronutrients are commonly observed in AITD and contribute to thyroid dysfunction.^11^

### Experimental Models and Environmental Factors

Animal studies have explored links between subclinical hypothyroidism (SCH) and gut microbiota imbalance. In murine models, SCH was induced using methimazole, while antibiotic perturbation assessed the effects of dysbiosis on thyroid function. Although alpha diversity and the Firmicutes/Bacteroidetes ratio showed no major differences between SCH and control mice, beta diversity revealed notable shifts. Fourteen bacterial genera significantly correlated with serum lipid levels, indicating a relationship between microbial imbalance, thyroid function, and dyslipidemia. Antibiotic-induced dysbiosis exacerbated hypothyroidism, underscoring the gut microbiota’s role in thyroid regulation.^8^^,^^12^

Environmental exposures, particularly PM2.5 (fine particulate matter with an aerodynamic diameter ≤2.5 μm), also shape the gut–thyroid axis. In Sprague–Dawley rats exposed to PM2.5, thyroid follicular epithelial damage, necrosis and hyperplasia were observed. 16S rRNA sequencing demonstrated reductions in Cyanobacteria, Bacteroidetes, and Proteobacteria, while metabolomic profiling revealed disruptions in thyroid hormone synthesis as well as tryptophan, glutathione, and histidine metabolism. These findings indicate that PM2.5 exposure exerts thyroid toxicity by altering gut microbial composition and metabolic pathways.^6^^,^^13^^,^^14^

Specific bacterial taxa, particularly Parabacteroides, correlated with metabolites such as glutathione and tryptophan derivatives, correlated with metabolite alterations, suggesting that PM2.5-induced thyrotoxicity is mediated through gut microbial and metabolic dysregulation. PM2.5 exposure also affected thyroid function markers (total T3, total T4, TSH) and reduced hypothalamic TRHR (thyrotropin-releasing hormone receptor) expression, while altering microbial diversity (increased Shannon index, decreased Simpson index). At the phylum level, Bacteroidota dominated (~90%), yet notable decreases in Cyanobacteria, Bacteroidetes, and Proteobacteria were observed in a dose-dependent manner. Additionally, genera such as *Elusimicrobium,* *Muribaculum*, *Eubacterium*, and *Parabacteroides* correlated with metabolites including taurocholic acid, glutathione, citric acid, and kynurenic acid.^8^^,^^10^^,^^14^

Shin et al^15^further investigated the effects of thyroid dysfunction on the gut microbiota using three rat models: levothyroxine-induced hyperthyroidism, propylthiouracil (PTU)-induced hypothyroidism, and surgical thyroidectomy. Distinct microbial shifts were observed across models. For instance, hyperthyroid rats showed an increase in *Ruminococcus*, while hypothyroid rats exhibited increased S24-7 family abundance but reduced *Prevotella*. Thyroidectomy markedly elevated Firmicutes and *Oscillospira*. These taxa are functionally relevant to host metabolism and immunity, as many members are involved in complex carbohydrate fermentation, short-chain fatty acid production, particularly butyrate, and modulation of mucosal immune responses, processes that may influence systemic inflammation and thyroid hormone homeostasis.

Hypothyroidism in animals, primarily mice or rats, can be induced pharmacologically by prolonged administration of PTU or methimazole, which inhibit thyroid hormone synthesis, leading to decreased serum T3 and T4 levels and a compensatory rise in TSH. Alternatively, surgical thyroidectomy produces permanent hypothyroidism, characterized by markedly reduced serum hormone levels, making this model particularly suitable for studying the long-term effects of thyroid hormone deficiency. Hyperthyroidism is induced through administration of levothyroxine (T4) or, occasionally, T3 injections, resulting in elevated serum thyroid hormone levels and suppression of TSH secretion, thereby reproducing hyperthyroid phenotypes. Moreover, autoimmune thyroid disease models, such as HT, can be generated by immunizing animals with thyroglobulin in combination with an adjuvant, eliciting an autoimmune response against thyroid tissue and elevated antithyroid antibody levels.^16^

### Gut–Thyroid Axis and Autoimmunity

Autoimmune thyroid conditions such as HT and GD have been linked to microbiota dysbiosis. Increased intestinal permeability may enhance systemic exposure to thyroid antigens and trigger autoimmunity. Clinical studies have associated hypothyroidism with SIBO and hyperthyroidism with distinct alterations in gut microbial structure. Both conditions are marked by microbial compositional changes. Hypothyroidism is often associated with bacterial overgrowth and microflora imbalance, whereas hyperthyroidism is linked to shifts in microbial diversity and genus-specific abundance.^5^^,^^8^

The thyroid–microbiota interaction occurs through several mechanisms:

- Micronutrient absorption: Gut microbiota regulate intestinal uptake of iodine, selenium, and iron, which are critical for thyroid function.
- Hormonal metabolism: Microbiota can influence the enterohepatic recycling of thyroid hormones.
- Immune modulation: Dysbiosis impairs barrier function and induces hyperactive immune responses, driving autoimmune processes.

Disruption of microbial balance may compromise intestinal barrier function, facilitating the systemic dissemination of microbial-derived molecules and promoting immune polarization toward proinflammatory pathways, a hallmark of autoimmune thyroid conditions.^7^^,^^8^

Thus, gut microbiota disturbances are intricately and bidirectionally linked to thyroid function and autoimmune diseases. Microbial imbalance not only initiates thyroid dysfunction but can also exacerbate disease progression. This field holds considerable promise for the development of microbiota-based targeted therapies in the future.^8^

### Clinical Evidence in Autoimmune Thyroid Diseases

Systematic reviews and meta-analyses of AITD cohorts (n = 196 patients across 8 studies) have demonstrated altered alpha diversity and differential microbial abundance compared with healthy controls. *Bifidobacterium* and *Lactobacillus* were consistently reduced, whereas potentially pathogenic taxa such as *Bacteroides fragilis* were increased. Notably, microbial richness (Chao1 index) was elevated in HT but decreased in GD. Other commensals such as Bacteroidetes and Lachnospiraceae were found in higher proportions in AITD patients.^17^

Leaky gut plays a central role in AITD pathogenesis. Translocation of inflammatory molecules like lipopolysaccharides may trigger aberrant immune responses to thyroid antigens. Short-chain fatty acids (SCFAs), especially butyrate, regulate Th17/Treg balance and support immune homeostasis. In AITD, reduced Treg counts and heightened Th17 activity disrupt this balance. Changes in the balance of these bacteria may influence the onset and severity of AITD through mechanisms such as Treg/Th17 imbalance, increased intestinal permeability, and increased systemic inflammatory burden.^20^

Microbial taxa associated with autoimmune disease activity are shown in Table 1. In HT, decreased alpha and beta diversity, reduced beneficial taxa (Lactobacillus, Bifidobacterium), and enrichment of Prevotella, Bacteroides, and Ruminococcus have been observed. These microbial changes may facilitate autoimmune responses to thyroid antigens by increasing intestinal permeability. Furthermore, leaky gut in HT may contribute to the rise of antithyroid antibodies. In GD and Graves’ orbitopathy, microbial shifts include altered Firmicutes/Bacteroidetes ratios and increased *Proteobacteria*. Microbial changes correlated with the severity of orbital inflammation, disease severity, inflammatory cytokines, and TSH receptor antibody levels. For example, the presence of certain bacterial species has been correlated with proinflammatory cytokine levels. It is thought that microbiota changes may be linked to levels of autoantibodies, such as TSH receptor antibodies.^21^^,^^22^

Treatment studies suggest that levothyroxine and antithyroid drugs influence the microbiota indirectly by restoring hormonal balance. However, thyroid hormones themselves may directly affect microbial composition, with hyperthyroidism linked to reductions in *Lactobacillus* and *Bifidobacterium*.^23^

### Future Perspectives and Therapeutic Implications

Understanding the gut–thyroid axis offers promising avenues for microbiota-targeted therapies. Probiotics, prebiotics, dietary interventions, and targeted supplementation (e.g., selenium, vitamin D) may restore immune balance and thyroid homeostasis. Animal models of hypothyroidism, hyperthyroidism, thyroidectomy, and induced autoimmune thyroiditis provide insights into mechanisms and therapeutic targets.^15^ ^24,^^25^

The interplay between gut microbiota, genetic predisposition, and environmental triggers contributes to the initiation and maintenance of autoimmune thyroid processes. Modulating gut microbial composition represents a potential adjunctive strategy to conventional therapies. Microbiota profiling may also serve as a biomarker for predicting disease activity, treatment response, and complications such as Graves’ orbitopathy.^7^^,^^23^ ^-^^25^

The gut–thyroid axis constitutes a dynamic, bidirectional network linking microbial ecology, endocrine regulation, and immune tolerance. Dysbiosis contributes to thyroid dysfunction via micronutrient malabsorption, immune dysregulation, and barrier impairment, while thyroid hormones reciprocally influence microbial composition. Therapeutic modulation of the microbiota holds significant potential for improving prevention, treatment, and long-term outcomes in thyroid disorders.^16^ ^24,^^25^

## Conclusion

In light of growing evidence, the gut microbiota is emerging not only as a bystander but also as an active participant in the pathogenesis of autoimmune thyroid disease. Specific microbial signatures, marked by decreases in beneficial taxa such as *Bifidobacterium* and *Lactobacillus* and increases in proinflammatory species such as *Collinsella* and *Eggerthella*, reflect immune system dysfunction seen in conditions such as HT and GD. Future research should aim to define causality, identify microbial biomarkers of disease activity, and evaluate the efficacy of microbiota-focused interventions such as probiotics, dietary modulation, or fecal microbiota transplantation. Ultimately, integrating microbiome science into endocrine practice may offer new strategies for personalized and preventive care in thyroid autoimmunity.

## Figures and Tables

**Table 1.** Microbial Taxa Associated with Autoimmune Disease Activity^18^ ^-^^20^

**Taxon**	**Correlation Type**	**Proposed Mechanism/Role**
Collinsella	Positive	Increased in Graves’ disease (GD); associated with proinflammatory cytokines and autoantibodies
Eggerthella	Positive	Linked to systemic inflammation and autoimmunity
Enterococcus	Positive	Associated with gut barrier disruption and inflammation
Ruminococcus gnavus	Positive	Activates proinflammatory pathways
Bifidobacterium	Negative	Supports immune tolerance; promotes regulatory T cell (Treg) induction
Lactobacillus	Negative	Protective against autoimmune responses
Faecalibacterium prausnitzii	Negative	Produces short-chain fatty acids (SCFAs); maintains Treg/Th17 balance
Roseburia	Negative*	Anti-inflammatory; however, its increased abundance in GD has unclear implications

*Roseburia shows anti-inflammatory properties, but its increase in Graves’ disease (GD) has uncertain significance.

## Data Availability

The data that support the findings of this study are available on request from the corresponding author.
